# The "Genius Loci" and the Patient

**Published:** 1912-06-15

**Authors:** 


### THE "GENIUS LOCI" AND THE PATIENT.

Sir William Osler, who sometimes, like one or two other ornaments of the profession, is in the position of a Platonist talking to Aristotelians and Lucretians, and sometimes speaks more from experience, not very long ago threw out a remark which we have since been waiting, with pleasant but so far vain anticipation, to hear him elaborate. It had to do with the remedial, or at any rate desirable, influence of the impression made upon the institutional patient by the prestige of the new, strange, specially adapted system of which he sees himself becoming a small part. This spell of the *genius loci* was alluded to by Professor Osler in connection with sanatoriums, but, of course, it takes a very wide range indeed.

A piece of news once overheard from a passer-by was as follows: "'E 'ave two 'ospitals, side by side, one for the rich and one for the poor, and the rich one pays for the poor one." Baseless, no doubt, it was, but one could not mistake the impressed tones of the ex-patient, or the effect it made on his companion. Under the spell, too, are the middle-class man, who, half amused and half conscious of self-denial, makes up his mind to do without whiskey and soda at the hydropathic, or the rich one who bows before the freaksome routine at Continental baths. Even the famous American brothers Mayo do not owe all their success to professional skill; the fine institutional organisation they have established must be reckoned a factor too. Two types with plenty of self-assurance are the police constable and the reporter; but see either mounting the hospital staircase and met with suave yet businesslike inquiry by the ward sister, and the *genius loci* has them quite subdued and diffident. The second-year medical student, who thinks nothing now of the dissecting-room, can hardly walk upon the unfamiliar slippery floor, with its foliaceous central table, for fear of tumbling down and causing some dire mischief.

The tendency of human nature we are trying to observe cuts, however, both ways. Successful treatment at home is nothing like so impressive as successful treatment in hospital, it is true; but if the doctor and nurse who have been called in do not please, the patient and his friends are not nearly so much upset as over falling foul in a similar way of an institution. Where the general practitioner is merely a demon, the hospital surgeon and house surgeon are (Mr. Bernard Shaw, and before him, Peter Mark Roget, are thanked for the word) cacodemons. This is why journals with no reputation, and no real ammunition either, find it easy to get mud to stick to hospitals. This, why hospital patients are so easily set off grumbling about their food; it is just in unconscious antagonism to their new subjection. Though they may have become a directed unit, yet they will show that for all that they know "what's what"; that they, too, are persons having authority, even if it be only over the disposal of eighteen shillings a week.

But further applications of Professor Osler's doctrine must be left to each reader. If it is the work of a poet to invent circumstance, it should be the aim of the essay-writer (and of the speaker, be it added) to avoid laborious compilation of the same, suggesting rather than exhausting his subject. The great practical bearing must, of course, be mentioned in conclusion—that as hospital workers risk undeserved blame on the one hand, and sometimes earn respect cheaply on the other, it behoves them, in forming their attitude towards patients, to balance the one fact against the other: mindful always, too, that they are dealing with those handicapped by sickness and distress, and ofttimes by ignorance.

